# What Are You Worried About? Content and Extent of Worry in Autistic Adults

**DOI:** 10.1007/s10803-023-05963-2

**Published:** 2023-03-29

**Authors:** Melissa H. Black, Dana L. Greenwood, Jerome Choo Chen Hwa, Jacqueline Pivac, Jessica Tang, Patrick J. F. Clarke

**Affiliations:** 1 Center of Neurodevelopmental Disorders (KIND), Centre for Psychiatry Research, Department of Women’s and Children’s Health, Karolinska Institutet & Stockholm Health Care Services, Region Stockholm, Stockholm, Sweden; 2 Curtin School of Allied Health, Curtin University, Perth, WA Australia; 3 enAble Institute, Curtin University, Perth, WA Australia; 4 Cognition and Emotion Research Group, Curtin University, Perth, WA Australia

**Keywords:** Worry, Anxiety, Depression, Autism spectrum disorders

## Abstract

Autistic adults commonly experience anxiety and worry, although knowledge on how worry presents and the content, extent, and experiences among autistic adults is limited. A convergent parallel mixed-methods approach was used to explore the presentation and experiences of worry in autistic and non-autistic adults. Quantitative surveys were used to compare the content and extent of worry in autistic adults to non-autistic adults, with semi-structured interviews also conducted with autistic adults to gain a deeper understanding of the experiences, impacts and content of worry in autistic adults. Findings indicated that autistic adults demonstrated clinically significant levels of worry which were substantially higher than non-autistic adults. Autistic adults described worry as a cycle of negative thoughts impacting their daily life. Findings indicate that autistic adults may worry more than non-autistic adults, impacting on participation in activities of daily living, sleep, and mental health.

Autistic adults often face a range of life challenges, many of which may be exacerbated by mental health difficulties that in turn can negatively affect quality of life (Lawson et al., 2020). That autistic individuals have been shown to have more than double the suicide mortality rate compared to the non-autistic population (Hwang et al., 2019) speaks to the level of psychological distress experienced by this population. It is important to understand the impact of the many unique challenges faced by autistic individuals and the processes that potentially contribute to and maintain psychological distress.

Mood and anxiety disorders such as generalised anxiety disorder, social anxiety, obsessive–compulsive disorder, panic disorder, and depressive disorders are common co-occurring conditions in autism (Hollocks et al., 2019; Lever & Geurts, 2016; Rosen et al., 2018). While anxiety disorders are often characterised by a fear or anticipation of future perceived threats, depressive disorders are characterised by pervasive low mood, diminished interest in or pleasure from activities, and disturbances in cognitive functioning (American Psychiatric Association, 2013). The pooled lifetime prevalence of anxiety disorders in autistic individuals is estimated to be 42%, while the lifetime estimate of an autistic individual experiencing a depressive disorder is 37% (Hollocks et al., 2019). Actual prevalence rates may however be under-estimated, given that diagnostic overshadowing may lead to symptoms of emotional disorders being instead attributed to characteristics of autism (Spain et al., 2018).

A common cognitive process known to be associated with many emotional disorders is worry. Worry is a core and defining feature of anxiety and is believed to be a common mechanism contributing to depression (Diefenbach et al., 2001), as well as other psychiatric conditions (Barlow et al., 2016; Ehring & Watkins, 2008). Though worry in small amounts is perceived to be potentially useful, and transient (Sweeny & Dooley, 2017), worry that is excessive and difficult to control can be damaging (Barlow, 2004). This pathological worry is exhibited as frequent repetitive negative thoughts about the future and the prospect of potential danger that becomes difficult for the individual to control or separate, and is often distressing for the worrier (Barlow, 2004; Goodwin et al., 2017; Hirsch & Mathews, 2012). Common content of pathological worry includes deep or considered thoughts about future (e.g., potential misfortunes and how they might be avoided) or past events (e.g., the implications of past mistakes or social missteps; Hirsch & Mathews, 2012).

In non-autistic populations, patterns of repetitive negative thinking are associated with poorer mental health and well-being (Watkins, 2008). Even in non-clinical populations, worry is associated with poorer mental health outcomes (Buck et al., 2008; Vîslă et al., 2022). Some evidence also suggests that engagement in worry has the capacity to prolong physiological activation to stress (Brosschot et al., 2006) and may increase risks to cardiovascular health (Tully et al., 2013), sleep disturbances (Kelly, 2018), somatic disorders, and ill-health (Brosschot et al., 2006; Watkins, 2008).

While poor mental health is highly prevalent in autistic adults, very few studies have sought to describe the presentation of worry in autistic adults. Previous research has however examined related concepts including rumination, perseveration, intolerance of uncertainty and cognitive inflexibility. Rumination and worry are overlapping concepts, thought to both relate to the over-arching cognitive processes of ‘repetitive negative thinking' (McEvoy et al., 2013). A key distinction, however relates to their temporal focus (Hoyer et al., 2009; Watkins et al., 2005). While worry is concerned with thoughts about future peril and negative outcomes typically associated with elevated anxiety, rumination is associated with thoughts focused on ones’ distress regarding symptoms, experience, or past negative events which are more commonly characteristic of depressed mood (Nolen-Hoeksema, 1998; Hoyer et al., 2009). Research examining rumination in autistic individuals has found that trait rumination is associated with depressive symptoms, similar to the non-autistic population (Williams et al., 2021). Autistic individuals also demonstrate a greater intolerance to uncertainty (i.e., tendency to react negatively to uncertain situations; Jenkinson et al., 2020), difficulty disengaging attention (Pugliese et al., 2015), and differences in cognitive flexibility (Fujino et al., 2017) that may place autistic individuals at a greater risk of worry. For example, some research has found that cognitive inflexibility is associated with measures of anxiety and depression in autistic adolescents and young adults (Hollocks et al., 2021).

Importantly, however, no study to date has specifically sought to describe the nature of worry in autistic adults. As such, the content, extent, and experiences of worry in autistic adults are largely unknown. Given that worry is a key internal cognitive process that could readily underlie the relationship between specific life challenges (work, social situations etc.), mental health, and psychiatric conditions such as anxiety and depression, there is a need to explore worry in autistic populations to determine whether its presentation is similar to neurotypical populations. For this reason, this study sought to identify the commonalities and differences in the content and extent of worry between autistic and non-autistic adults, and to describe the specific experiences of worry in autistic adults.

## Methodology

### Design

A convergent parallel mixed-method design was used to explore the content, extent, experiences, and impact of worry among autistic adults, allowing deeper exploration of the specific factors influencing worry and to what extent this impacts their lives (Creswell & Creswell, 2017; Creswell & Plano Clark, 2011). In a convergent parallel mixed-method design, both quantitative and qualitative data collection and analysis occur separately and simultaneously before interpretations are merged to provide a greater understanding of the phenomenon than can be offered by a single method alone (Creswell & Creswell, 2017). In this study, a quantitative online survey examined the self-reported content and frequency of worry in autistic and non-autistic adults while a concurrent qualitative methodology, using semi-structured interviews, provided detailed insights into content, experiences, and impacts of worry and coping strategies implemented.

### Participants

To better capture the experiences of the general autistic and non-autistic population, a broad community recruitment approach was undertaken. Participants were recruited via autism-specific services, social media, and a database of participants (including those with autism) who had previously expressed their interest in participating in a broad range of research related to autism. Participants for the quantitative survey included autistic and non-autistic adults. Inclusion criteria for both autistic and non-autistic participants included the following: (1) aged 18 years and above (2) resided in Australia for the past 2 years and (3) completed 80% or more of the survey. Further inclusion criteria for autistic participants included; (1) a diagnosis of autism with no co-occurring intellectual disability according to DSM-IV-TR (American Psychiatric Association, 2000) or DSM-5 (American Psychiatric Association, 2013) from a health professional confirmed via self-report or self-diagnosed. The decision to include self-diagnosed individuals was in recognition of barriers that individuals may face to obtaining a diagnosis and the fact that previous research has found that self-diagnosed individuals resemble individuals with a formal diagnosis across several domains (McDonald, 2020). Autistic adults with common co-occurring conditions such as Attention Deficit Hyperactivity Disorder (ADHD), anxiety, and epilepsy were included in the study. Non-autistic participants were excluded if they (1) scored above 32 on the Autism Spectrum Quotient indicating high autistic-like traits (Woodury-Smith et al., 2005). A total number of 274 individuals participated in the survey, comprising of 54 autistic adults and 220 non-autistic adults. Seventeen participants (two autistic and 15 non-autistic) were excluded due to incomplete survey completion. A further ten non-autistic participants were excluded due to scoring above the cut-off score on the AQ. Analysis therefore included 52 autistic and 195 non-autistic participants. Autistic and non-autistic groups did not differ on age (*t* (245) = 0.911, *p* = 0.363), however there was a significant difference between the two groups for gender (*X*^*2*^ (1, *N* 244) = 4.378, *p* = 0.036) reflecting a higher proportion of female participants in the non-autistic sample. Due to differences in gender, analyses were also conducted accounting for gender as a covariant, however this showed no effect on the significance or direction of any results. For this reason, analyses without covarying for gender are presented.

Participants in the qualitative component consisted of 14 autistic adults. Inclusion criteria included the following, (1) a diagnosis of autism with no co-occurring diagnosis of intellectual disability according to DSM-IV-TR (American Psychiatric Association, 2000) or DSM-5 (American Psychiatric Association, 2013) as confirmed via self-report or self-diagnosed, (2) able to communicate verbally, (3) proficient conversational English, (4) aged 18–65 years. Participants in the qualitative component were recruited concurrently and seperately from the quantitative component through social media, autism-specific services, and a database of autistic individuals who had previously participated in research. Prior to data collection it was estimated that between 6 and 15 participants would be required to reach data saturation. This estimation was based on qualitative interviews conducted in similar areas (Cheak-Zamora & Odunleye, 2022; Halim et al., 2018; Spain et al., 2020). Following each interview, researchers carefeully reflected upon and discussed key discussions arising from each interview and how they added to an undertsanding of the phenomenon, with data collection ceasing at participant 14. Demographic characteristics for participants in the qualitative component are displayed in Table 1.

**Table 1:** Demographic data statistics of autistic and neurotypical participants

	Quantitative survey autistic N = 52		Quantitative survey non-autistic N = 195		Qualitative interview autistic N = 14	
	N	%	N	%	N	%
Age mean years (SD)	38.19 (14.3)		31.21 (54.7)		40.5 (16.5)	
Gender						
Male	21	40.4	52	26.7	9	64.3
Female	29	55.8	142	72.8	4	28.6
Non-Binary	2	3.9	1	0.5	1	7.1
Diagnosis						
Confirmed	50	96.2	-	-	13	92.9
Self-diagnosed	2	3,9	-	-	1	7.1
Diagnosis Type						
Autism Spectrum Conditions	29	58	-	-	8	57.1
Autistic Disorder	3	6.0			-	-
Asperger’s Disorder	18	36			6	42.9
Psychiatric Conditions*						
Anxiety disorders	16	30.8	14	7.2	8	57.1
Depressive disorders	17	32.7	12	6.2	8	57.1
Trauma- and stressor-related disorders	7	13.5	0	0	2	14.3
Other neurodevelopmental conditions (e.g., ADHD)	3	5.8	5	2.6	1	7.1
Obsessive–compulsive and related disorders	2	3.8	2	1.0	1	7.1
Feeding and eating disorders	1	1.9	1	0.5	0	0
Schizophrenia spectrum and other psychotic disorders	1	1.9	0	0	0	0
Medications for psychiatric conditions						
SSRIs	8	15.4	6	3.1	2	14.3
Tricyclic antidepressants	6	11.5	0	0	1	7.1
SNRIs	6	11.5	0	0	1	7.1
Mood stabilizers	1	1.9	0	0	0	0
Antidepressants unspecified	3	5.8	3	1.5	1	7.1
Anti-anxiety agents	4	7.7	0	0	3	21.4
Anxiety unspecified	2	3.8	0	0	0	0
Stimulants	2	3.8	3	1.5	0	0
ADHD unspecified	1	1.9	0	0	0	0
Antipsychotic unspecified	1	1.9	0	0	0	0
Atypical antipsychotics	1	1.9	1	0.5	2	14.3

*ADHD* Attention Deficit Hyperactivity Disorder, *SD* Standard Deviation, *SSRI* Selective serotonin reuptake inhibitor antidepressants, *SNRIs* Serotonin-norepinephrine reuptake inhibitor antidepressants

*Conditions as self-reported by participants, categorized according to DSM-5

### Materials

#### Quantitative Survey

##### Autism Spectrum Quotient (AQ)

The AQ was used to measure autistic-like traits in five domains; (1) social skills, (2) communication skills, (3) imagination, (4) attention to detail and (5) attention switching/tolerance of change (Woodury-Smith et al., 2005). The 50-item questionnaire is scored on a four-point scale, where scores of zero to 25 indicate ‘few or no autistic traits’, 26 to 31 indicate ‘mild autistic traits’ and 32 to 50 indicate ‘clinically significant levels of autistic traits’ (Woodury-Smith et al., 2005). The AQ has good reliability and validity (Woodury-Smith et al., 2005).

##### Depression Anxiety and Stress Scale–21 (DASS-21)

The DASS-21 is a short form version of the DASS, measuring self-reported states of depression, anxiety and stress (Lovibond & Lovibond, 1995). It consists of 21-items rated on a four-point scale. The DASS-21 has demonstrated good internal consistency and adequate validity for autistic adults (Park et al., 2020).

##### Worry Domains Questionnaire (WDQ)

The WDQ is a 25 item self-reported questionnaire that gives an indication of worry frequency and impact on everyday life and was scored using a five-point scale, with higher scores equating to greater levels of worry (Tallis et al., 1992). The WDQ is scored on five subscales exploring worry experienced in (1) relationships, (2) lack of confidence, (3) aimless future, (4) work and (5) financial (Tallis et al., 1992). The five domains in the WDQ are scored individually to obtain a total score for each sub-scale in addition to a combined score of zero to 40 (Tallis et al., 1992). The WDQ has good reliability and validity in non-autistic samples (McCarthy-Larzelere et al., 2001).

##### Penn-State Worry Questionnaire (PSWQ)

The PSWQ measures clinically significant trait worry exploring the dimensions of generality, control and excessiveness of worry (Meyer et al., 1990). It contains 16 items scored on a five-point scale. The scale is scored by summing all items with responses to questions one, three, eight, 10, and 11 reverse scored (Meyer et al., 1990). Total scores range from 16 to 80 with higher scores indicating greater worry. Scores ranging 52–65 indicate that individual has some problems with worry that may benefit from treatment, with a score of 66 or above indicating that worry is chronic and requires treatment (Meyer et al., 1990). The PSWQ has good reliability and validity with highly satisfactory internal reliability in non-autistic samples (Rijsoort et al., 1999).

##### Dunn Worry Questionnaire (DWQ)

The DWQ is a short 10-item measure scored on a four point scale (0 = none of the time, 4 = all of the time) whereby participants rate their experiences of worry over the preceding one month period. The DWQ is designed to measure general problematic worry, including items focused on control of worry and its emotional impact. The 10 items of the scale are summed with a maximum total score of 40, with scores over 21 being indicative of clinically high levels of worry (Freeman et al., 2020).

#### Additional Questions

As worry is an under-researched area in autism, and because there exists no psychometrically evaluated worry questionnaires for autistic adults, additional questions were asked after the worry questionnaires to capture possible areas of worry not covered in the questionnaires.

#### Qualitative Interview

A semi-structured interview guide co-designed with an autistic researcher and autistic adults, sought to explore experiences and impacts of worry, including how autistic adults feel they experience worry, how these worries manifest in the body, and the overall impact this has on their lives (Table 2).

**Table 2:** Semi-structured interview guide questions

Number	Question
1	What does worry mean to you?
2	Do you experience worry?
3	What does worry feel like to you (for example in your body, your mood)?
4	What are the types of things you worry about?
5	How much do you think you worry? (how frequently? how intense?)
6	Are there specific times (e.g., of the day) that you worry more?
7	Are there specific situations that cause you to worry more?
8	How does worry impact/affect you?

### Procedure

#### Quantitative Survey

The online survey was administered via Qualtrics (Qualtrics, Provo, UT). Participants were provided with information about the study via a participant information sheet and were required to provide consent via the online platform prior to beginning the survey. An open-ended question was included at the end of the survey; giving participants the opportunity to provide further details on anything they specifically worried about that may not had been captured in the questionnaires.

#### Qualitative Interview

Semi-structured interviews using open ended questions were used to obtain qualitative data to gather a subjective view of the nature, experience, and impact of worry. Participants who provided written informed consent received a copy of the interview guide prior to the interview. Participants were provided with the option of engaging in a face-to-face (*n* = 3), telephone (*n* = 6), or online (*n* = 5) interviews to reduce anxiety and uncertainty related to the interview process (Howard et al., 2019). All interviews were audio-recorded.

### Data Analysis

#### Quantitative Survey

Data collected via the survey were cleaned and imported into SPSS version 26.0 for analysis (IBM Corporation, 2019). Demographic data were explored using descriptive statistics (Table 1) and examined to determine if groups were comparable. Independent samples t-tests were performed to determine differences between groups on the PSWQ, WDQ and DWQ total scores. Where Levene’s test was significant, degrees of freedom were adjusted, and due to the uneven sample size, hedges g was used to provide a measure of effect size. As the WDQ consists of five subdomains, the WDQ was submitted to a mixed model Analysis of Variance (ANOVA) with group as the between subject factor (autistic and non-autistic), and WDQ domains as the within subject factor (Relationships, Lack of Confidence, Aimless Future, Work Incompetence, and Financial). Partial Eta squared (ηp2) were used to report effect size estimates.

#### Qualitative Interview

Audio recordings were transcribed verbatim and checked for accuracy prior to being imported into NVIVO 12 software for analysis (QSR International, 2018). Thematic analysis was then undertaken by the research team following the six steps outlined by Braun and Clarke (2006). First researchers familiarised themselves with the data by reading the transcriptions. During this process, initial notes were taken of ideas arising within the transcripts. Transcripts were then discussed by the research team and notes were compared. Each transcript was then re-read with codes developed for each data extract. The coding focused on identifying latent and semantic features within the data describing the phenomenon. A primarily inductive approach was taken to code development with the aim of ensuring analysis captured the experiences of participants, however, elements of a deductive approach were also employed to ensure that themes could be integrated with the quantitative data (Braun & Clarke, 2021). Identified codes were subsequently examined for areas of similarity and divergence and codes were grouped into themes. The resulting themes were then reviewed by the research team and named. Throughout data analysis, the research team debriefed regularly to ensure the credibility of the research (Nowell et al., 2017). All members of the research team collaborated to improve the rigour of the data via written reflexive journals, as well as multiple members being involved within the data analysis and coding. All aspects of this study were clearly documented following a logical system to provide an audit trail (Nowell et al., 2017). To ensure that findings were an accurate representation of participants’ responses member-checking was conducted both during the interviews and following analysis. Within the interview, interviewers sought to confirm their understanding of the participant responses, and to clarify areas of possible ambiguity in interpretation (Lincoln & Guba, 1985). After thematic analysis, participants also received the researchers’ interpretation of the interviews and feedback was sought on the thematic analysis to confirm that interpretations were clearly derived from the participants’ data (Lincoln & Guba, 1985; Nowell et al., 2017). Participants who provided feedback confirmed that findings were an accurate representation of their experiences, one participant provided additional examples and explanation which were integrated into the themes.

#### Integration

**Fig. 1:**
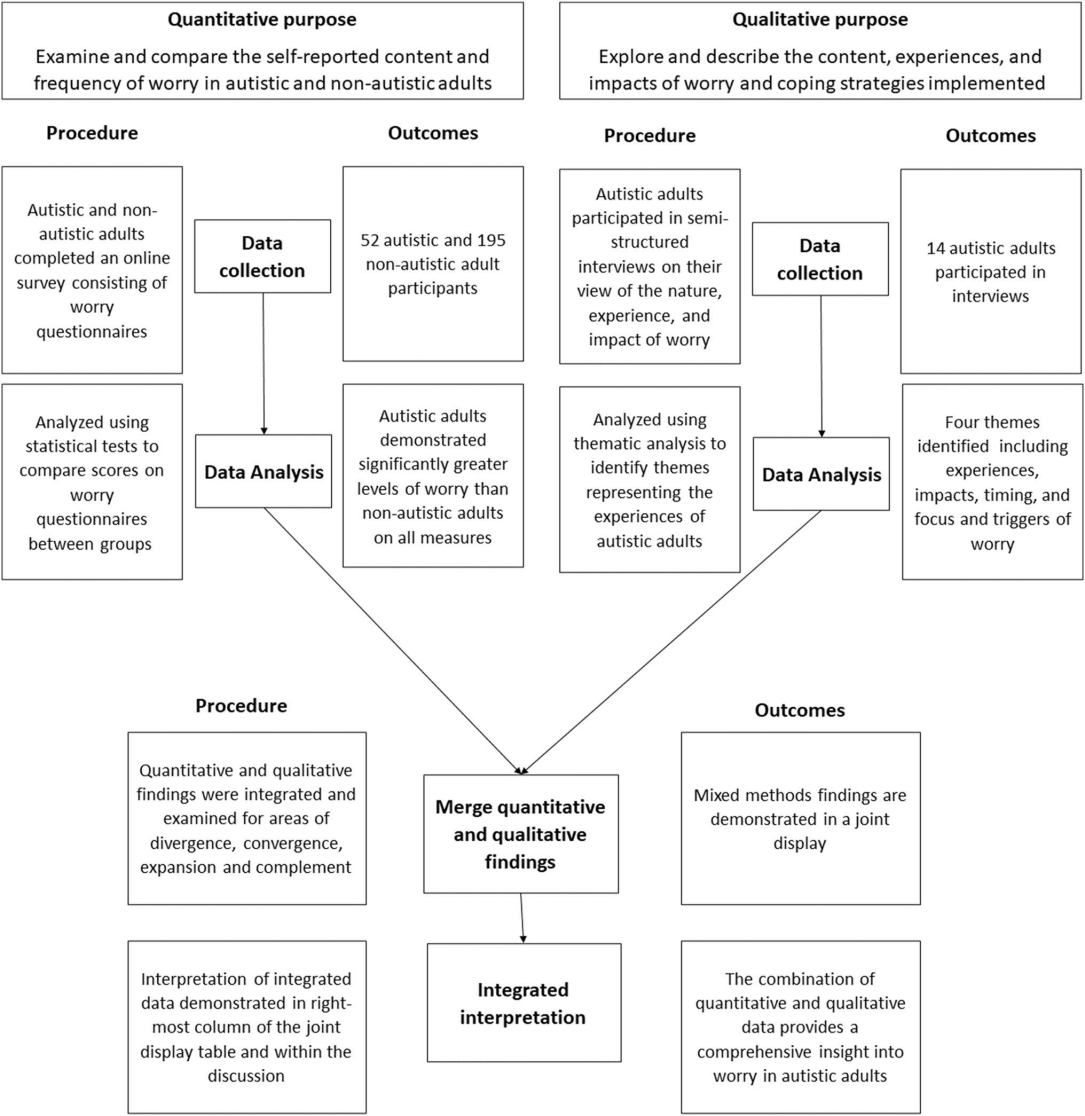
Visual depiction of convergent parallel mixed-method design

The mixed-method process and integration of qualitative and quantitative findings is displayed in Fig. 1. Quantitative and qualitative data were integrated and examined for areas of divergence, convergence, expansion and complement (Fetters, 2019). In accordance with Fetters (2019), convergence refers to areas where the two streams agree, divergence refers to areas where quantitative and qualitative findings are contradictory, complementary refers to areas where the streams differ but do not contradict each other, and expansion refers to areas where the streams largely converge, but with opportunity for additional interpretation. To assist in integration of findings, a joint display table (Creswell & Creswell, 2017) was developed.

### Ethical Considerations

Ethical approval was obtained from the Human Research Ethics Committee (HRE2020-0768) at Curtin University in Perth, Western Australia. Pseudonyms were used to ensure confidentiality. As a token of appreciation for their time, survey participants were provided with the opportunity to enter a randomised draw to win one of three $50 gift cards, and all interview participants received a $10 gift card.

## Results

### Quantitative Results

Autistic adults demonstrated significantly greater levels of worry than non-autistic adults as measured by the total score of the PSWQ, *t* (69.719) = 4.763, *p* < 0.01, *g* = 0.883). When examining the distribution of scores, 53.8% of autistic adults scored above 66, consistent with the criteria for ‘chronic worry’. By comparison, 26.7% of non-autistic adults registered a score consistent with this criterion. Similarly, autistic adults demonstrated significantly greater worry on total scores of the WDQ, *t* (70.603) = 5.406, *p* < 0.001, *g* = 0.940), and DWQ* t* (243) = 4.785, *p* < 0.001,* g* = 0.745) (Table 3).

**Table 3:** PSWQ, DWQ and WDQ comparisons between groups

	Autistic	Non-autistic
PSWQ mean (SD)	71.71(24.38)	54.27(19.68)
DWQ mean (SD)	30.96(7.02)	25.49(7.40)
WDQ—total mean (SD)	75.08(23.73)	55.73(19.59)
WDQ—relationship mean (SD)	14.39(5.53)	10.39(4.76)
WDQ—lack of confidence mean (SD)	16.67(5.75)	12.53(5.35)
WDQ—aimless future mean (SD)	15.65(5.98)	11.04(4.73)
WDQ—work mean (SD)	15.23(5.58)	11.79(4.31)
WDQ—finance mean (SD)	13.13(5.25)	9.98(3.9)

*PSWQ* Penn State Worry Questionnaire, *DWQ* Dunn Worry Questionnaire, *WDQ* Worry Domain Questionnaire

**Fig. 2:**
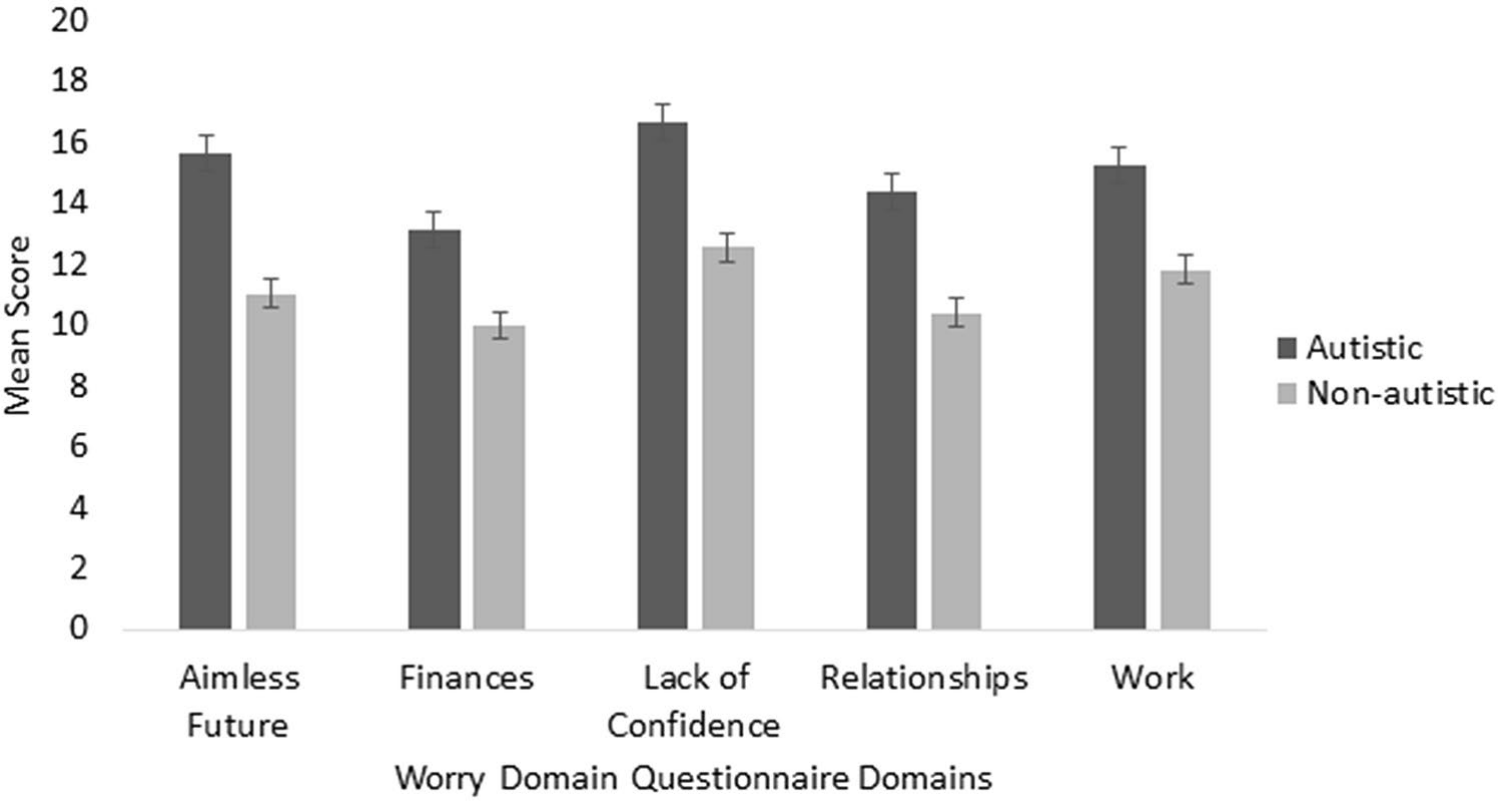
Autistic and non-autistic ratings on Worry Domain Questionnaire domains. Error bars represent ± 1 standard error

The mixed model ANOVA exploring differences between the groups on the WDQ (Table 3 and Fig. 2) found a main effect of group, *F*(1) = 36.467, *p* < 0.001, ηp2 = 0.130), indicating that autistic adults had significantly greater worry across all WDQ domains when compared to non-autistic adults. A main effect of domain was also found, *F* (3.306) = 26.025, *p* < 0.001, indicating that regardless of group, levels of worry across domains differed. Post-hoc comparisons showed that regardless of group, participants exhibited higher scores in the domain of lack of confidence compared to all other domains (all *t* > 3.768, *p* < 0.001, g > 0.239). Participants tended to worry less about relationships compared to all other domains (all t > −10.846, p < 0.01, g > −0.339), except for financial (t (246) = 1.941, *p* = 0.053). Significant effects for the total sample effects were also observed between aimless future and financial (t (246) = 4.719, p < 0.001, g = 0.300), and work and financial (t (246) = 6.222, p < 0.001, g = 0.395) participants reporting higher general worry about the future and work more than finance. No interaction effects were found.

When comparing autistic and non-autistic groups on emotional factors relating to psychological distress using the DASS, a main effect of group *F*(1,220) = 50.709,*p* < 0.01), and an interaction effect between DASS scale and group was found *F*(1.923,432.061) = 5.735,*p* < 0.01, nP2 = 0.187), indicating that autistic adults had greater, stress, anxiety and depression than compared to non-autistic adults.

In autistic adults, stress scores showed a significant positive correlation with scores on the WDQ (*r* = 0.686, *p* < 0.01), PSWQ (*r* = 0.417, *p* < 0.01) and DWQ (*r* = 0.786, *p* < 0.01). Anxiety scores showed a significant positive correlation with the WDQ (*r* = 0.656, *p* < 0.01) and DWQ (*r* = 0.683, *p* < 0.01), while depression scores also correlated with the WDQ (*r* = 0.686, *p* < 0.01) and DWQ (*r* = 0.627, *p* < 0.01). No relationships were found between anxiety or depression scores on the DASS with the PSWQ (*r* < 0.189, *p* > 0.192). In non-autistic adults, stress, depression and anxiety scores on the DASS showed significant positive correlations with the WDQ, PSWQ and DWQ (all r > 0.450, *p* < 0.001).

### Other Worries

When asked if there was anything that not covered in the questionnaires that participants worried about, 25 (48%) autistic adults provided additional worries. Some autistic adults who were caregivers reported being worried about their children and their parenting, including being able to advocate for their child and how their health might affect their children. Autistic adults also worried about their relationships, including whether they were holding their partners back or how day-to-day misunderstandings could affect their relationships. Some also worried about being judged by others, particularly neurotypicals. Additional worries were shared by 51 (26%) of non-autistic adults (Table 4).

**Table 4:** Additional worries shared by respondents

Autistic adults	n (%/ 52)	Non-autistic adults	N (%/195)
That I'm not a good parent/that I won’t be able to advocate for my child	6(12)	That my family is safe and health	11(6)
My relationships	5(10)	Health and fitness	5(9)
People will know that I'm different and judge me/treat me differently	4(8)	Losing relationships/my relationships	3(5)
That I will let people down	4(8)	Performance in education/employment/work	3(5)
Everything	3(6)	What others think of me	4(2)
That my life has no purpose/I have no personality	3(6)	My future	4(2)
My health/what will happen when I can't care for myself	3(6)	Money	2(1)
Traffic and driving	2(4)	Pets	2(1)
My future	2(4)	World events	2(1)
		Appearance	2(1)
		Making an impact/achievement	2(1)

Only topics with more than one respondent are reported

### Qualitative Results

Thematic analysis of autistic adults participating in interviews identified four themes, including Experiences of Worry, Impacts of Worry, Timing of Worry, and Focus and Triggers of Worry.

### Experiences of Worry

The majority of participants described worry as constant negative thoughts and feelings. Jasmine shared that “[worry was] a sort of hyper-focusing on something and not really being able to think about anything else”. Stephanie similarly shared that “…when I’m really worried about something it’s kind of like my brain becomes separate from my body, so I’ll not notice things about my body because I’m totally in my head. You know, it just takes over.”

Worry was described as a never-ending cycle involving overthinking, where thoughts circulated through participants’ minds throughout their daily lives. Shaun expressed that “… worry can be a rabbit warren of fears…”. This idea was common throughout the interviews, which were reported to lead to self-doubt, constant fear, and over-thinking. One participant explained that being an over-thinker caused them to “examine everything with a fine toothcomb” (Elizabeth). Another participant, Jane, related this excessive focus on thoughts to them being “… a really deep thinker and always has been… I worry about things that people probably don't even ever consider.”

### Impacts of Worry

Worry was reported by participants to substantially affect their ability to participate in daily life, including the ability to function and perform tasks necessary for living such as cooking, attending medical appointments, and accessing the community. Participants reported that worry impacted their lives by decreasing their mood, increasing their fear of socialising, and influencing their relationships with family and friends. One participant highlighted this impact on daily life, as it was “easy to lose track of other things… it can be a risk of getting into more negative thought patterns” (Jeff). While other participants shared how worry led to them being “less able to focus…”, “…less able to think positively”, being “… tense” and “… not taking in information…” Stephanie discussed how worry felt:

*I find things that I would normally just do automatically quite difficult, I trip over things, I drop things, I just can’t concentrate, I feel tired, …my muscles hurt. I get pain...like, I am so absorbed in my head, I forget everything*

Worry was found to reduce participants accessing their community and was reported to contribute to health and financial issues, job instability, and overall affecting their ability to remain independent and support themselves. Due to the worry experienced, most participants stated that they found it very difficult to remain independent and function in day-to-day tasks. Worry experienced particularly during the evenings impacted participants’ abilities to sleep, with Jake stating, “I do find if I am worried about stuff. I'll have days when I just can't sleep, or I'll maybe sleep for about four hours a day or less…”.

Worry was seen to decrease when coping strategies such as writing their thoughts down in a journal, pre-planning the day before, or reading a book as a distraction were adopted to reduce worry. As one participant stated, “trying to put some sort of relaxation techniques in place … trying not to be on my phone before bed and things like that … having a book that I can read, just to shut off for a little bit” (Jasmine).

### Timing of Worry

Participants most commonly reported that worry increased throughout the day feeling as though “… it gets worse throughout the day” and getting “…worse over time” (Lachlan). Particularly higher levels of worry were reported when attempting to fall asleep, “…when I’m lying in bed at night … that’s one time when I do worry a bit more than other times” (Shaun), and immediately after waking up in the morning “it can take a bit longer to get started in the morning” (Jasmine). These situations were reported to increase worry as participants found that they became more aware of their thoughts due to a lack of other distractions, and they began to interact within the ‘worry cycle’. As one participant stated, “…if you’re lying there in bed, then, of course, you’re more acutely aware of your thoughts because your body’s not doing anything” (Daniel). Some participants described particular times of the day relating to daily activities, such as dinnertime and after work, having an impact on increasing their levels of worry. In contrast, one participant reported heightened levels of worry only “…once every two to three days” (Levi).

### Focus and Triggers of Worry

Participants expressed that they worried about a range of factors (Table 5). The majority of participants reported that they worried about social situations and their relationships. In particular, participants reported worrying about “doing something wrong” socially or that they “hadn’t done enough for someone”.

**Table 5:** Focus of worry described by autistic participants

Area of worry	N (%)
Uncertain or new situations	9(64)
The future	8(57)
Family	8(57)
Finance	7(50)
Potential social missteps/social interactions	7(50)
Uncertain or new situations	7(50)
Own health	4(29)
World events	4(29)
Personal safety	3(21)
Worry about work/getting work	3(21)

Uncertain situations also worried many participants, with one participant describing this as the “uncertainty factor”. For example, participants worried about who they would see in the community or what would “happen to them while they were outside of the house”. Meeting new people and “planning what was going to happen” that day were also sources of stress. Other examples included participants reporting worrying about whether a package they had ordered would arrive that day or what they would cook for dinner. Tasks that needed to be completed were also a source of worry for some participants, such as needing to book an appointment.

Participants also reported worrying about the health of their family and their own health. Connor shared: “…Family concerns about how your kids are, about health in general, whether it's mine, or whether it's anyone in the family”. Other common worries included worrying about finances and job performance and to day life. Seth reported that he worried about “… driving, going out to the shops, meeting new people … and planning [their] day”. Future events such as “when will I get my own house”, “when will I have enough income” or “what will I be doing” were also discussed. Some participants reported an increase in worry associated with worldwide events including the COVID-19 pandemic. In addition, participants stated the uncertain nature of these events and unpredictable changes affect their normal routine.

### Integration of Qualitative and Quantitative Findings

Table 6 presents the joint display table showing the meta-inferences and interpretations arising from the integration of quantitative and qualitative data. Three overarching themes were identified from the integration of data, (1) levels of worry, (2) impact of worry and (3) focus of worry. The overarching theme “levels of worry” showed evidence that qualitative and quantitative data were complementary, with both streams demonstrating that autistic adults experience worry that can be troubling to them and difficult to control. Areas of expansion could also be observed, with the qualitative findings adding insight into the feelings associated with high levels of worry in autistic adults. Evidence of expansion was observed in the overarching theme “impact of worry”. Quantitative findings from the DWQ highlight the emotional impact of worry on participants, while the qualitative interviews provide additional description of the impact of worry, particularly in participation in daily life and functioning. The final overarching theme “focus of worry” showed both areas of convergence, as well as divergence. Areas of convergence can be observed in the areas of worry identified, with both quantitative and qualitative findings highlighting some similarities in the focus of worry (i.e., finances, future, relationships, work, confidence). However, areas of divergence were also identified, with autistic adults in the qualitative study describing worries more specifically about social missteps and uncertainty that may represent unique experiences of worry for autistic adults.

**Table 6:** Joint display table showing integration of quantitative and qualitative findings with meta-inferences and interpretation

Overarching theme	Quantitative finding	Qualitative finding	Meta-inferences and interpretation
Levels of worry	Autistic adults demonstrated significantly greater worry than non-autistic adults on the PSWQ, WDQ and DWQ	Autistic adults described worry as constant and difficult to escape from	Complementary: Autistic adults experience worry that is troubling to them and difficult to control Expansion: Qualitative findings add insight into the feelings associated with high levels of worry
Impact of worry	Autistic adults demonstrate significantly greater worry on the DWQ compared to non-autistic adults	Autistic adults described that worry impacted across all domains of life and reduced participation	Expansion: The DWQ demonstrates the emotional impact of worry, while qualitative data describes the impact of worry on daily functioning and participation
Focus of Worry	Autistic adults demonstrated significantly greater worry on all domains of the WDQ	Autistic adults described a broad range of areas of worry (Table 5)	Convergence: Similar areas of worry identified across qualitative and quantitative data Divergence The qualitative study identified worries not captured in the quantitative data, specifically about social missteps and uncertainty

## Discussion

This study provides a holistic investigation of the content, extent, and experiences of worry in autistic and non-autistic adults. Through employing a convergent parallel mixed-method design, this study found that autistic adults worried significantly more than non-autistic adults across all worry domains, reported to significantly impact their ability to participate and engage in daily life across a number of areas.

Autistic adults showed levels of worry that may be considered pathological or problematic. Trait worry in autistic adults was observed to be high, with the average score indicating that the sample had worry that could benefit from treatment. Importantly, half (53.8%) of autistic adults scored above 66, consistent with the criteria for ‘chronic worry’ compared to 26.7% of non-autistic adults in the sample based on cut-offs for the PSWQ (Meyer et al., 1990). Quantitative and qualitative findings show that while worry was greater in autistic adults than compared to non-autistic adults, the content and focus of worries was largely congruent with those of non-autistic adults. Consistent with results from the PSWQ, data from the WDQ and DWQ indicated that the autistic adults worried significantly more than the non-autistic adults overall. Comparison across the individual worry domains covered in the WDQ further suggested that such worry was not disproportionately concentrated in specific domains, with consistently higher worry reported across all areas, and no evidence of an interaction between worry domain and group.

Autistic adults reported being worried about how others would perceive them and whether they would fit in, with worry perceived to be particularly exacerbated by social situations including engaging with people in the community. The exacerbation of worry in regard to social situations may, in part, be resultant of the known social communicative differences of this population (American Psychiatric Association, 2013). Autistic adults may have had previous negative social experiences, contributing to increased concern about their performance in social settings. Indeed, autistic children face higher levels of isolation and bullying (Humphrey & Hebron, 2015), with exclusion known to follow autistic individuals into adulthood (Jones et al., 2022). For some autistic adults, evaluations of social performance may have begun as adaptive attempts to evaluate and improve performance, which have turned maladaptive because of frequent negative interactions. This was also re-enforced by some autistic adults reporting that they worried about receiving judgement from others, particularly neurotypical individuals. While assisting autistic adults to develop neurotypical social skills may play a role in promoting positive interactions across development, and thus, potentially reducing worry related to social interactions, conceptualising social skills from a purely neurotypical perspective (i.e., that one particular way of communicating is the ‘correct’ way) may act to worsen worry about ones’ own performance. Resultantly, consideration of how social skills training is conceptualized and providing strategies that promote inclusion and acceptance are required.

Alongside worries about social situations, autistic adults reported worry about new and uncertain situations. A preference for routine or ‘sameness’ is a core diagnostic criterion for autism. It has been proposed that insistence for sameness may be a self-regulatory strategy that attempts to maximise predictability in the environment to avoid unexpected or anticipated fears (Uljarević et al., 2017). Such a self-regulatory strategy has been observed in neurotypical children, but has been proposed to be retained in autism, despite no longer being helpful, potentially resulting in anxiety (Uljarević & Evans, 2017). Relatedly, a desire for predictability may be contributing to worry in autistic adults where they are uncertain of the environmental demands that may be present in a situation and how they may be controlled. This may therefore be another area of worry more specific to autistic adults.

Consistent with the possibility that autistic adults may experience worry in a number of areas not covered by traditional instruments (such as the WDQ), 48% of autistic adults detailed additional worries not covered in the questionnaires compared to 26% of the non-autistic adults. Of particular relevance was the observation that many of the additional worries described by autistic adults relate to concerns that are unique to autistic individuals. For example, while non-autistic adults described general worries for their children’s welfare (“I worry about them crossing the road”), the worries of autistic parents often focused on the impact of their autism on their children (e.g. “I worry that I won’t be able to advocate for my children”, “I worry that my mental health will affect my child”). This was also the case for interpersonal concerns where autistic adults worried that their autism may contribute to them doing or saying something that could cause them to lose friends or offend others. Collectively, these observations highlight that there are a number of areas that may be the source of specific worry for autistic adults, and importantly, that these are unlikely to be captured by traditional worry measures.

While this study did not seek to examine the factors that may underlie the development and maintenance of worry in autistic adults, some hypotheses may be drawn from what is known about worry in non-autistic adults. Cognitive models of worry in non-autistic populations propose that those predisposed to worry may have differences in bottom-up and top-down attentional control (Hirsch & Mathews, 2012). That is, that worriers may have negative emotional processing biases, appraising ambiguous information as more threatening, and may have reduced capacity to exert top-down efforts to control and constrain negative representations of threat (or perceived threat) (Hirsch & Mathews, 2012). Indeed, autistic adults had worry that more was more difficult to control than non-autistic adults as measured by the DWQ (Freeman et al., 2020). Difficulties with cognitive control have been reported in autistic individuals previously (Mackie & Fan, 2016), and therefore a reduced cognitive control capacity in autistic individuals may increase their susceptibility to worry. On the other hand, worry can also diminish the finite control and attentional resources available to an individual, potentially contributing to the differences in cognitive control observed in autistic individuals. Differences in cognitive flexibility (monotropism) may also provide a pathway through which autistic adults are more vulnerable to worry, with some research suggesting that cognitive inflexibility may be associated with internalising symptoms indirectly through enhanced uncertainty (Ozsivadjian et al., 2021; South & Rodgers, 2017). Unknown or uncertain events are more likely to be perceived negatively or threatening if tolerance to uncertainty is low (Dugas et al., 2001). For those with high intolerance to uncertainty, worry may pose as a (maladaptive) coping strategy in attempts to gain additional control over a situation (Dugas et al., 2001). Intolerance to uncertainty is commonly observed in autistic individuals and a link between anxiety and intolerance to uncertainty in autism has been established (Jenkinson et al., 2020).

While such dispositional qualities may contribute to heightened levels of worry, the present research also highlights that autistic adults are likely to experience a number of unique stressors that could serve to elevate worry and anxiety. The notion that specific groups of individuals may experience stress as a function of their specific minority status in society is one that has been increasingly investigated (Meyer, 1995). This has been specifically recognised in relation to ethnic minority groups (Rehman et al., 2021), and LGBTQIA + populations (Weeks et al., 2021), but pressures to fit with specific societal norms and/or conceal aspects of oneself has also been applied to autistic populations (Botha & Frost, 2018). It may therefore be important for future research to develop instruments capable of indexing specific aspects of worry and stress experienced by autistic individuals to better understand and address issues that are not shared with the broader community upon which most instruments are normed and validated.

The concerning levels of worry reported by autistic adults in the current study also speak to the need for tailored interventions to reduce worry. Worry is a transdiagnostic process and may therefore benefit from targeted intervention regardless of the formal diagnoses held by an individual (McEvoy et al., 2013). Interventions may target worry specifically, but also its core underlying components such as intolerance to uncertainty (Jenkinson et al., 2020). To aid in the development of effective interventions; research is also required to examine processes operating in autism that may make autistic individuals particularly vulnerable to worry. In particular, as attention and cognitive control mechanisms are known to function differently in autism (Hirsch & Mathews, 2012), establishing the contribution of these mechanisms to the development and maintenance of worry is required. Future research may also investigate what protective factors and coping strategies may make autistic individuals more resilient to worry.

### Limitations

The following limitations should be taken into consideration. This project was undertaken within the context of COVID-19, with high amounts of negative emotions markedly worry, evident within the general population of Australia (Rossell et al., 2021). The pandemic may have increased worry levels in both autistic and non-autistic adults, but it is unknown if this had a disproportionate impact on autistic adults compared to non-autistic adults which could have skewed results. Further, though efforts were made to recruit a broad representative sample, it is possible that participants with greater levels of worry opted to participate in the study, potentially biasing results. This research was conducted within the Australian demographic and as such not all aspects of worry will necessarily generalise across populations. As participation required a level of intellect and English comprehension, the results will not necessarily be representative of the entire Australian autistic adult population, particularly for those with intellectual disability who did not take part. A screening of intellectual abilities and a formal assessment of worry and anxiety were not undertaken, further limiting generalisability. It should also be noted that as there are currently no worry questionnaires validated for use with the autistic population, we selected measures that are well established in non-autistic individuals. The WDQ, DWQ and PSWQ have not been used within the autistic population and do not contain any specific questions for autistic people, therefore it is unclear whether the questionnaires were as reliable or valid for use with autistic individuals, as it is unknown if autistic participants interpreted questions similarly to non-autistic participants. For this reason, the results must be interpreted with caution. To counter this during both the survey and the interviews, participants were asked an open-ended question regarding if there was anything else they worried about to capture these potentially unique experiences. Future research may benefit from validating existing worry questionnaires, adapting questionnaires, or developing new autism-specific worry questionnaires to more accurately capture the experiences of worry in this population.

## Conclusion

This study explored the content and extent of worry experienced among autistic adults compared to non-autistic adults. Autistic adults worried more than non-autistic adults in every aspect of daily living, with autistic adults reporting this led to more isolating behaviours. Concerns relating to the uncertainty of future events and potential misfortune were consistent themes. Participants highlighted a range of areas where they believed worry has a significant impact on daily functioning including sleep, positive relationships, future planning, financial wellbeing, and general health. While autistic adults were found to worry more overall, questionnaire findings did not suggest that the pattern of worry differed significantly from non-autistic adults. However, autistic adults did describe areas of worry that were not covered in quantitative questionnaires that appear unique to this population. Such findings highlight the potential importance of future research developing population specific measures that have the capacity to capture aspects of worry unique to autistic adults. This will assist in tailoring interventions to addressing debilitating worry in autistic adults.
